# Gliricidia leaf meal and multi-enzyme in rabbits diet: effect on performance, blood indices, serum metabolites and antioxidant status

**DOI:** 10.1186/s40781-018-0182-8

**Published:** 2018-10-06

**Authors:** Olugbenga David Oloruntola, Johnson Oluwasola Agbede, Simeon Olugbenga Ayodele, Eyanlola Soladoye Ayedun, Olajumoke Temidayo Daramola, Deborah Adebukola Oloruntola

**Affiliations:** 1 Animal Science Department, Adekunle Ajasin University, Akungba Akoko, Nigeria; 2 Animal Production and Health Department, The Federal University of Technology, Akure, Nigeria; 3 Department of Agricultural Technology, The Federal Polytechnic, Ado Ekiti, Nigeria; 4 Microbiology Department, The Federal University of Technology, Akure, Nigeria

**Keywords:** Leaf meals, Rabbits, Exo-enzyme, Performance, Health status

## Abstract

**Background:**

Following the ban on the importation of import-dependent fed ingredients in most developing countries, the need to look inward for local content is now compelling. Thus, leaf meals that have phytogenic additive potentials are envisaged will be a viable feed ingredient in rabbit diets.

**Methods:**

The effect of dietary inclusion of gliricidia leaf meal (GLM) with or without multi-enzyme (E) supplementation in rabbits was investigated using ninety-six 35-day old rabbits of crossbreed (Newzealand and Chinchilla). One basal diet that met the requirements of growing rabbit was formulated (Diet 1). Thereafter, another two diets were formulated to contain 15% GLM and 15% GLM plus multi-enzyme at 1 g/kg and designated as diets 2 and 3 respectively. The rabbits were randomly distributed into the 3 diets (32 rabbits/treatment; 4 rabbits/replicate) and fed their respective experimental diets for 8 weeks.

**Results:**

The body weight and daily weight gain of the rabbits fed on GLM free diet and those on GLM-based diets (diets 1 and 2) were similar at finishing period of 63–91 day but have lower (*P* < 0.01) values than those rabbits fed GLM + E based diet (diet 3) at finishing period (63–91 days) and whole fattening period (35-91 days). The apparent dry matter and crude protein digestibility of rabbits fed control diet and those fed 15% GLM based diet were lower (*P* < 0.05) than those fed 15% GLM + E-based diet. Triglycerides concentration of rabbits fed 15% GLM-based diet without enzyme addition were lower (*P* < 0.05) than those observed for rabbits on the rest test diets. Cholesterol and Low-Density Lipoprotein levels of rabbits fed 15% GLM and 15% GLM + E-based diets were lower (*P* < 0.05) than those fed the GLM free diet. The superoxide dismutase and glutathione peroxidase of rabbits fed the GLM free diet (diet 1) were significantly (*P* < 0.05) lower than those fed the 15%GLM and 15% GLM + E-based diets.

**Conclusion:**

Dietary inclusion of GLM at 15% of the diet did not have a negative effect on the rabbits postweaning period (35–63 days) but will require multi-enzyme supplementation to enhance growth indices at finishing period (63–91 day) without precipitating negative effect on the rabbits’ health status.

## Background

The use of plants in different forms as alternatives to the relatively expensive and scarce conventional feed resources in the production of monogastric animals is becoming more popular in the tropics. This is because these plants and their parts could serve as an indispensable source of protein, phytobiotics and antioxidants in monogastric nutrition [1, 2]. Some phytochemicals in plants improve antioxidant, anti-microbial, feed flavour and palatability which could result in increased feed intake and performance in animals [3]. These tropical plants are available because of their rapid growth which is enhanced by the prevailing and environmental factors. Their dietary inclusion in form of meals is capable of reducing the cost of commercial feeds, with a resultant reduction in the cost of animal protein and improvement in animal health [2, 4, 5].

Gliricidia (*Gliricidia sepium*) provide large quantities of high-quality biomass all year round; contain high-quality protein and minerals in adequate concentration [5, 6]. Gliricidia leaves have also been reported to contain compounds such as flavonoids, polyphenolics and saponin [7] which could serve as drawbacks/feed ingredients in compounding their use as animal feeds at relatively high inclusion level. However, the addition of exogenous enzymes is envisaged could help alleviate the deleterious effects of these secondary metabolites an animal [8], improve nutrient availability [9], reduce ileal flow and mortality of the rabbits [10].

The effects of nutrition on performance traits and haematological traits [11, 12] and the general wellbeing of an animal cannot be underscored. Effect of some herbs on general performance, nutrient utilization, antioxidants, haematological indices and serum enzymes in monogastric animals have been documented [2]. However, report on the effect of gliricidia leaf meal on growth indices, blood and serum metabolite as well as the antioxidant status in the rabbit is rear when the diet is supplemented with exo-enzyme. The later thus formed the basis for this study.

## Methods

### Experimental site

The experimental procedures involving animals were approved by the Agricultural Technology Department Research and Ethics Committee, The Federal Polytechnic, Ado Ekiti, Nigeria. This study was carried out at the Livestock Unit of the Teaching and Research Farm of The Federal Polytechnic, Ado Ekiti, Nigeria. The experimental site is 437 mm above sea level and on latitudes 7^o^37^o^N and longitude 5^o^11^o^E, with the annual temperature of 26.2 °C and the annual rainfall of 1247 mm [13].

### Preparation of leaf meal and experimental diets

Fresh gliricidia leaves were harvested within the precinct of The Federal Polytechnic, Ado Ekiti, Nigeria. The leaves were air dried for 3 weeks and milled using hammer mill (3.0 mm sieve) to make Gliricidia leaf meal (GLM). Thereafter, the GLM was analyzed for the proximate composition using the methods of AOAC [14] and tannin using the procedure described by Markkar and Goodchild [15]. The gross energy was determined with combustion calorimeter (Model:e2k combustion calorimeter, www.cal2k.com). The commercial enzyme (Biozyme PH) used was procured from a reputable vendor in Ado Ekiti, Nigeria. It was manufactured by Biomix S.A, Carrera 47C, Sabaneta-Colombia and has a minimum of cellulase (700,000 U.A), phytase (1,200F.T.U), protease (8,000,000 U.P), α-amylase (800,000 U.A), β-glucanase (300,000 U.BG), lipase (20,000 U.I) and xylanase (500,000 U.X). A basal diet (Diet 1) was formulated to meet the minimum requirement of a growing rabbit (16.88% crude protein, 17.59% crude fibre and 2670.52 Kcal/kg Metabolizable energy). Another diet was formulated to include 15% GLM and balanced for crude protein, crude fibre and metabolizable energy. This diet was thoroughly mixed and divided into two portions. The first portion was designated Diet 2 while the second portion was supplemented with 1 g/kg of the commercial enzyme and designated Diet 3. All the diets were pelletized (4 mm in diameter and 8 mm long). The compositions of the experimental diets are shown in Table 1.

**Table 1:** Composition of experimental diet (%)

	Diet 1 0%GLM	Diet 2 15%GLM	Diet 3 15%GLM + E
Ingredients (%)			
Maize	8.00	6.00	6.00
Wheat offals	8.00	7.00	7.00
Soybean meal	16.10	10.80	10.80
Maize husk	22.00	21.00	21.00
Gliricidia leaf meal	0.00	15.00	15.00
Cassava peels	22.00	20.00	20.00
Brewers dried grain	21.70	18.00	18.00
Bone meal	1.10	1.10	1.10
Premix	0.25	0.25	0.25
Methionine	0.20	0.20	0.20
Lysine	0.10	0.10	0.10
Salt	0.25	0.25	0.25
Vegetable oil	0.30	0.30	0.30
Determined analysis (%)			
Crude protein	16.88	16.91	16.89
Crude fibre	17.59	17.93	17.95
Neutral detergent fiber	39.18	38.91	38.95
Acid detergent fiber	17.59	17.22	1726
Acid detergent lignin	3.68	6.65	6.62
^a^Metabolizable energy (Kcal/kg)	2670.52	2670.56	2670.56

^a^Calculated analysis

### Animal, housing and experimental procedure

Ninety-six, 35-day old weaner rabbits (32 rabbits/ treatment; 4 rabbits/replicate) of cross-breed (New-Zealand and Chinchilla) of equal sexes and weighed 675.03 ± 8.02 g were randomly distributed to 3 dietary treatments. The rabbits were housed in galvanized wire meshed cages (60x60x30 cm) in a well-ventilated pen and fed ad libitum throughout the experimental period (56 days) with their respective experimental diets. The rabbits were managed in line with the recommendations and guidelines for applied nutrition experiments in rabbits [16]. The body weight and feed consumption of the rabbits were determined and recorded on 7 days interval and used to calculate the daily weight gain and feed conversion ratio. Also, the mortality was checked daily. At 45 days into the trial, eight (8) rabbits were selected randomly from each dietary group and caged individually in metabolism cages (55x35x26 cm). The rabbits were adapted for 7 days and thereafter, hard faeces were collected from them daily into polythene bags at approximately 08:00 h each morning before serving daily ration for 4 days. The faeces were stored in a freezer (-18 °C) until chemical analysis. On day 55 of the experiment, 2 rabbits per cage (16 rabbits per diet) were selected, tagged, starved overnight, weighed and sacrificed (at approximately 8:00 h in the morning) as described by Blasco et al. [17]. The skin, limb and head were removed before evisceration of the carcass. The dressing weight and weights of the internal organs (lungs, kidney, liver, heart and spleen) were determined. Thereafter, the dressing percentage and the relative weights of the internal organs were calculated as the percentage of slaughter weight.

On day 56 of the experiment, blood samples were collected from the marginal ear vein of the rabbits (2 rabbits/cage; 16 rabbits/diet) as described by Brunett et al. [18]. Each blood sample collected (about 7 ml) was divided into two. One portion was placed in potassium ethylene diamine tetraacetic acid (KEDTA) sample tube (for haematology) and the other in the plain tube (for serology and antioxidant enzyme determination). Thereafter, Shenzhen Mind ray Auto Haematology Analyser (Model Bc-3200, Shenzhen Mind ray Biomedical Electronics Co. Hamburg 20,537, Germany) was used to determine the haematological indices while the serum chemistry indices were determined with a Reflectron ® Plus 8C79 (Roche Diagnostic, GonbH Mannheim, Germany) using kits. Glutathione peroxidase (GPx) activity and superoxide dismutase (SOD) in serum sample were determined as earlier described by Rotruck et al. [19] and Misra and Fridovich, [20], respectively. Catalase activity was also determined as previously described by Aebi, [21].

### Chemical and statistical analyses

The three diets were analysed for their crude protein and crude fibre as described by AOAC [14]. The neutral detergent fibre, acid detergent fibre and acid detergent lignin were also determined [22]. Data generated in the animal trial were analyzed using SPSS 2011 package (version 20) following standard procedure for analysis of variance. Statistical significance was assessed at (*P* < 0.05) while the means were separated using Duncan multiple range test of the same statistical package.

## Results

Table 2 shows that the body weight (BW), daily weight gain (DWG), daily feed intake (DFI) and feed conversion ratio (FCR) of rabbits fed the control diet (GLM free diet) were not significantly (*P* > 0.05) different from those fed the test diets at postweaning period (35-63d). However, the BW of the rabbits on the control diet and those on 15% GLM without exo-enzyme (E) supplementation were statistically similar but have lower (*P* < 0.01) value than those fed 15% GLM + E at finishing (63–91 days) and whole fattening period (35–91 days).

**Table 2:** Effects of Gliricidia leaf meal and exo-enzyme supplementation on performance traits of rabbits

	Diet 1 0% GLM	Diet 2 15%GLM	Diet 3 15%GLM + E	SEM	*P* value
No of rabbits	32	32	32		
Post-weaning period (35–63 d)					
Body weight at 35 d (g)	672.44	674.88	677.73	2.67	0.77
Body weight at 63 d (g)	1544.15	1582.90	1630.62	29.93	0.56
Daily weight gain (g/d)	31.13	32.42	34.03	1.01	0.57
Daily feed intake (g/d)	78.31	76.87	81.31	4.11	0.93
Feed conversion ratio	2.53	2.41	2.39	0.15	0.94
Finishing period (63–91 d)					
Body weight at 91 d (g)	2171.50^b^	2239.90^b^	2358.16^a^	29.42	0.01
Daily weight gain (g/d)	22.41	23.46	25.98	0.99	0.36
Daily feed intake (g/d)	82.53	82.19	90.31	5.44	0.83
Feed conversion ratio	3.70	3.56	3.52	0.27	0.97
Whole fattening period (35–91 d)					
Daily weight gain (g/d)	26.76^b^	27.94^b^	30.01^a^	0.51	0.01
Daily feed intake (g/d)	77.64	76.79	82.85	4.51	0.87
Feed conversion ratio	3.01	2.85	2.86	0.17	0.94

Means within a row with different letters are significantly different (*P* < 0.05)

*GLM* Gliricidia leaf meal, *E* exogenous enzyme

Table 3 shows that only apparent digestibility (AD) of the dry matter (DM) and crude protein (CP) were significantly (*P* < 0.05) influenced by the dietary treatment. For instance, while the apparent DM digestibility of rabbits fed the GLM free diet (64.30%) and 15% GLM–E (67.11%) supplemented diet were similar (*P* > 0.05), the AD of the DM of those rabbits fed 15% GLM were significantly (*P* < 0.05) lower (59.93%) than those fed 15% GLM + E diet but similar to those fed the GLM free diet (64.30%). The same trend was also observed for the apparent CP digestibility. In both the DM and CP apparent digestibility, rabbits fed on the 15% GLM + E diet consistently had the highest values with rabbits fed on the 15% GLM-based diet having the lowest values.

**Table 3:** Effects of Gliricidia leaf meal and exo-enzyme supplementation on dry matter (DM) intake and apparent digestibility (%)

	Diet 1 0% GLM	Diet 2 15%GLM	Diet 3 15%GLM + E	SEM	*P* value
No of rabbits	8	8	8		
Feed intake (g DM/d)	73.38	71.41	75.43	1.15	0.41
Dry matter	64.30^ab^	59.93^b^	67.11^a^	1.27	0.03
Crude protein	65.62^ab^	63.52^b^	66.32^a^	1.00	0.02
Crude fibre	28.99	28.58	30.26	0.92	0.79
Neutral detergent fibre	47.29	46.53	48.58	1.04	0.77
Acid detergent fibre	27.91	27.62	28.51	0.57	0.85

Means within a row with different letters are significantly different (*P* < 0.05)

*GLM* Gliricidia leaf meal, *E* exogenous enzyme

Also, Table 4 shows that the slaughter weight, hot carcass weight, commercial slaughter weight, liver, kidney, lung, heart and gallbladder of rabbits fed the GLM free diet and those fed the two test diets were not significantly (*P* > 0.05) influenced by the dietary treatment.

**Table 4:** Effects of Gliricidia leaf meal and enzyme supplementation on carcass and relative organ (% slaughter weight) of rabbits

	Diet 1 0% GLM	Diet 2 15%GLM	Diet 3 15%GLM + E	SEM	*P* value
No of rabbits	16	16	16		
Slaughter weight (g)	1953.80	1966.16	1943.65	10.69	0.74
Hot carcass weight	58.37	62.13	62.53	0.98	0.16
Commercial slaughter weight	16.59	15.28	14.41	0.86	0.65
Liver	3.29	3.19	3.40	0.04	0.09
Kidney	0.66	0.60	0.61	0.01	0.06
Lung	0.41	0.42	0.43	0.01	0.97
Heart	0.23	0.26	0.23	0.01	0.64
Gallbladder	0.07	0.04	0.06	0.01	0.33

Means within a row with different letters are significantly different (*P* < 0.05)

*GLM* Gliricidia leaf meal, *E* exogenous enzyme

The mean values of the various haematological indices of rabbits studied were not significantly (*P* > 0.05) different (Table 5), while the triglycerides, cholesterol and Low-Density Lipoprotein (LDL) values were significantly (*P* < 0.05) affected of the entire serum metabolites measured (Table 6). Serum triglycerides concentration of rabbits fed 15%GLM supplemented diet were lower (*P* < 0.05) than those observed for rabbit on the rest test diets. The serum cholesterol and LDL levels of rabbits fed 15%GLM and 15%GLM + E supplemented diets were lower (*P* < 0.01) than those fed the control. Table 7 shows the effect of GLM and enzyme supplementation on the oxidative status of the experimental rabbits. The superoxide dismutase and glutathione peroxidase of rabbits fed the GLM free diet were consistently significantly (*P* < 0.003, 0.01) lower than those fed the 15% GLM and GLM + E diets.

**Table 5:** Effects of Gliricidia leaf meal and exo-enzyme supplementation on haematological indices of rabbits

	Diet 1 0% GLM	Diet 2 15%GLM	Diet 3 15%GLM + E	SEM	*P* value
White blood cells (× 10^9^/l)	7.85	5.79	7.07	0.41	0.11
Monocytes (×10^9^/l)	0.58	0.49	0.49	0.07	0.86
Granulocytes (×10^9^/l)	4.17	3.74	3.46	0.16	0.22
Lymphocytes (×10^9^/l)	3.09	1.55	3.11	0.39	0.18
Red blood cells (× 10^12^/l)	5.93	6.44	6.01	0.16	0.44
Haemoglobin concentration (g/dl)	14.00	13.65	13.40	0.23	0.62
Packed cell volume (%)	39.46	39.96	38.11	0.81	0.69
MCV (fl)	65.50	62.00	62.67	1.22	0.31
MCH (pg)	23.65	21.15	22.13	0.58	0.23
MCHC (g/dl)	35.50	34.15	35.20	0.34	0.27

Means within a row with different letters are significantly different (*P* < 0.05)

*GLM* Gliricidia leaf meal, *E* exogenous enzyme, *MCV* Mean cell volume, *MCH* Mean cell haemoglobin, *MCHC* Mean cell haemoglobin concentration

**Table 6:** Effects of Gliricidia leaf meal and enzyme supplementation on serum metabolites of rabbits

	Diet 1 0% GLM	Diet 2 15%GLM	Diet 3 15%GLM + E	SEM	*P* value
Total protein (g/l)	57.80	59.50	61.55	1.17	0.48
Urea (μmol/l)	15.25	11.40	8.92	1.20	0.07
Triglycerides (mg/dl)	1.17^a^	1.10^b^	1.15^a^	0.01	0.03
Cholesterol (mg/dl)	5.62^a^	2.93^b^	3.09^b^	0.48	0.01
LDL (mg/dl)	4.44^a^	0.94^b^	1.70^b^	0.56	0.01
HDL (mg/dl)	0.49	0.43	0.57	0.03	0.27
Alanine aminotransferase (U/L)	92.20	60.55	49.60	8.61	0.09
Aspartate aminotransferase (U/L)	45.45	51.10	53.85	2.36	0.38
Bilirubin total (mg/dl)	11.45	10.52	12.40	0.48	0.32
Creatinine (mg/dl)	66.05	77.55	73.10	2.79	0.26

Means within a row with different letters are significantly different (*P* < 0.05)

*GLM* Gliricidia leaf meal, *E* exogenous enzyme, *LDL* Low-density lipoprotein, *HDL* High-density lipoprotein

**Table 7:** Effects of Gliricidia leaf meal and enzyme supplementation on oxidative status of rabbits

	Diet 1 0% GLM	Diet 2 15%GLM	Diet 3 15%GLM + E	SEM	*P* value
Superoxide dismutase (%)	22.71^b^	33.91^a^	40.48^a^	2.79	0.003
Glutathione peroxidase (μg /g)	62.26^b^	81.53^a^	89.17^a^	4.38	0.005
Catalase (mM/ml/min)	0.006	0.002	0.003	0.001	0.643

Means within a row with different letters are significantly different (*P* < 0.05)

*GLM* Gliricidia leaf meal, *E* exogenous enzyme

## Discussion

The similarity in the performance indices of rabbits fed 15% GLM with or without enzyme-based diets and the control diet at post-weaning period (35-63 days) in this study is a pointer to the possible suitability of gliricidia leaf meal as a good feedstuff which can be used as replacer to the import-dependent feed resources in rabbits’ diets at post-weaning period. The use of leaf meals in the formulation of monogastrics’ and or rabbit feeds had been identified as a helpful way of alleviating the problem emanating from competition between human and animals for some conventional feedstuff [6, 9, 23]. Furthermore, the superiority of the rabbits fed gliricidia leaf meal based diet supplemented with exogenous multi-enzyme in terms of higher body weight at day 91(finishing period: 63-91d) and daily weight gain during the whole fattening period (35–91 d) support the efficacy of the exo-enzyme to enhance the utilization of the nutrients (DM and CP) in the diet for better growth performance [24]. Dietary enzymes supplementation in monogastrics enhances the breaking down of compounds in animal feeds which may not be effectively broken down by the animals’ own digestive enzyme and thereby improve the productivity of animals. This is achieved following the disruption of cell-matrix of fibrous feedstuffs by the exogenous enzymes; thereby giving easy access of the endogenous enzymes to digest the entrapped nutrients [25]. This result is consistent with the report of Eiben et al. [26] but the contrast with a report of Ayodele et al. [9], who reported a non-significant effect of enzyme supplementation on performance of rabbits. The observed variations in the two reports can be attributed to many factors among which are the dietary component and age of the animals [9, 27] and the enzyme composition used in the trials. The observed lower dry matter (DM) and crude protein (CP) apparent digestibility recorded in rabbits fed on 15% GLM based diet (diet 2) when compared to those fed 15% GLM + E based diet (diet 3) might be due to the activities of secondary metabolites (anti-nutrients) and fibre which were not sufficiently biologically degraded; as the multi-enzyme used in the current study (diet 3) contained varying degrees of phytase, cellulose, protease, α-amylase, β-glucanase, lipase and zylanase activities of which their inclusion could result in increased anti-nutrients degradation and improved nutrient utilization [28]. In this study, the inclusion of 15%GLM led to 0.2 g tannin/100 g in the diets 2 and 3 (Table 8) and tannin has long been implicated in the reduction of protein utilization in animal [29]. The negative effect of this anti-nutritional factor seems to have been removed by the relatively higher crude protein digestibility value recorded in multi-enzyme supplemented diet and this is consistent with the current report of Ayodele et al. [9].

**Table 8:** Chemical composition (%) and energy (Kcal/kg) of Gliricidia leaf meal

Parameters	Quantity
Dry matter	93.03
Crude protein	24.75
Crude fibre	12.65
Ash	8.65
Ether extract	1.61
Nitrogen-free extract	45.37
Tannin	1.31
Energy	3840

Beyond reduced weight gain; animals have been reported to have an abnormal change in the weight and morphology of their internal organs as the response to toxicity situation. In this study, the slaughter weight, hot carcass weight, commercial slaughter weight and the relative weights of liver, kidney, lung, heart and gallbladder were similar across the treatment groups, suggesting that the inclusion of GLM and GLM + E promoted similar muscle and organ development and that the organ’s integrity of the rabbits was not compromised. This disagreed with an earlier report of Ayodele et al. [9], that the use of Alchornea leaf meal had an effect on carcass and relative weights of the organ of growing rabbits.

Nutrition has a strong influence on the haematological traits; and values of these haematological traits are indicators of the nutritional status of the animals [30–32]. In this study, values of all the haematological indices fall within the normal range [18] and were not different across the dietary groups. Contrary to the present report, Adeyeye et al. [31] reported a variation in the haemoglobin concentration, red blood cells and mean cell volume when processed cocoa pod husk meal was fed to rabbits. Oloruntola and Ayodele [32] also reported a variation in white blood cells, lymphocytes and platelets in rabbits fed 0% and 10% pawpaw leaf meal inclusive diet. However, this present study further confirms the nutritional adequacy of the diets used in this study and in particular indicating the safety of the treatment applied in supporting the normal haemapoietic process in the experimental rabbits. Elevated and or highly elevated triglyceride level may be a risk factor for atherosclerosis, pancreatitis and liver disease. In this study, reduced level of triglycerides in rabbits fed 15% GLM diet compared to the rest test diets signifies the possible presence of some bioactive compounds in GLM which impaired fat absorption and consequent fat depletion. This is further supported by the reduced cholesterol and Low-Density Lipoprotein level (LDL) in rabbits fed 15%GLM and 15%GLM + E- based-diets compared to the control diet in this study as this is also of health benefit to the consumers, especially those predisposed to heart disease. The inclusion of GLM in the diets vis-a-vis decreased uptake of cholesterol or increased loss or catabolism of cholesterol [33] further confirms the health benefits of including GLM in rabbit’s diet. The observed decreased in cholesterol level in this study may be linked to the presence of saponins, one of the components of gliricidia which exerts inhibitory effects on cholesterol uptake in the gut through intra-lumenal physiochemical interaction [29]. However, the present findings agreed with earlier reports of Oloruntola et al., [30]; Adeyeye et al. [31] and Oloruntola and Ayodele [32], who all reported a reduced cholesterol level in rabbits fed *Alchornea cordifolia* leaf meal based diets, processed cocoa pod husk meal based diet and pawpaw leaf meal inclusive diet, respectively.

One of the major causes of retarded growth in livestock is oxidative stress. However, the use of herbal based antioxidants to ameliorate the stress is becoming popular [34]. Antioxidants significantly delay or prevent oxidation of lipids, protein, carbohydrates and DNA [35]. For instance, an antioxidant enzyme like glutathione peroxidase, superoxide dismutase and catalase can prevent oxidation either by stabilizing transition metal radicals such as Fe^2+^ or Cu^+^ or by scavenging initiated free radicals such as superoxide and hydrogen peroxide, the most reactive free radical in vivo [36]. In this study, the 15% GLM and 15% GLM + E based diets were found to promote the scavenging of superoxide ion by increasing superoxide dismutase activity. In the same trend, the higher plasma values of glutathione peroxidase, a catalyst for various peroxides degradation in rabbits fed 15% GLM and 15% GLM + E based diets compared to the control further revealed the antioxidant properties of the leaf meal in use in this study. This further confirms the use of GLM as safe feed ingredients in the diets of growing rabbit.

## Conclusion

Dietary inclusion of GLM at 15% of the diet had no effect on rabbits’ performance at post-weaning period (35–63 day) but requires the use of multi-enzyme like the one used in this study to enhance performance at finishing period (63-91 days) and whole fattening period (35–91 d) without deleterious effects on the dry matter and crude protein digestibility. The 15% GLM dietary inclusion with or without multi-enzyme had no deleterious effect on the rabbits’ carcass cut, organ’s integrity and the haematological indices; while triglyceride, cholesterol and LDL were reduced and the oxidative status of the rabbits was enhanced. Thus, the use of GLM + E could help to stem the overdependence on import duty feed ingredients used in rabbit feed formulation in this sub-Sahara Africa where the shortage of animal protein is endemic.

## Abbreviations

AD: Apparent digestibility
AOAC: Association of Analytical Chemist
BW: Body weight
CP: Crude protein
DFI: Daily feed intake
DM: Dry matter
DWG: Daily weight gain
E: Multi-enzyme
FCR: Feed conversion ratio
GLM: Gliricidia leaf meal
GPx: Glutathione peroxidase
KEDTA: Potassium ethylene diamine tetraacetic acid
LDL: Low density lipoprotein;
SOD: Superoxide dismutase
SPSS: Statistical Package for Social Sciences
